# Quality Assessment of Cookies Made from Composite Flours Containing Malted Barley Flour and Wheat Flour

**DOI:** 10.3390/plants11060761

**Published:** 2022-03-12

**Authors:** Marko Jukić, Gjore Nakov, Daliborka Koceva Komlenić, Nastia Vasileva, Franjo Šumanovac, Jasmina Lukinac

**Affiliations:** 1 Faculty of Food Technology Osijek, Josip Juraj Strossmayer University of Osijek, 31000 Osijek, Croatia; dkoceva@ptfos.hr (D.K.K.); fsumanovac@ptfos.hr (F.Š.); jlukinac@ptfos.hr (J.L.); 2 Institute of Cryobiology and Food Technologies, Agricultural Academy—Sofia, 53 Cherni Vrah Blvd., 1407 Sofia, Bulgaria; gnakov@ikht.bg; 3 College of Sliven, Technical University of Sofia, 59 Bourgasko Shaussee Blvd., 8800 Sliven, Bulgaria; nastia_vas@tu-sofia.bg

**Keywords:** short-dough cookies, malted barley flour, sucrose reduction

## Abstract

Wheat-based short-dough cookies are considered low nutritional value foods because their recipes are high in fat and sugar. The aim of this study was to investigate the effects of replacing part of the wheat flour (WF) with different types of malted barley flour (MBF), while reducing sucrose in the recipe, in order to produce cookies with increased nutritional value, enhanced functional properties, and acceptable technological and sensory characteristics. Three types of brewer’s MBF (*Pilsen*, *Amber*, and *Black*) were used to replace WF in amounts of 20, 40, and 60%, while simultaneously reducing the addition of sucrose. Sucrose was added at levels of 66.6, 33.3, and 0% of the original standard recipe. MBF mitigated the effects of the reduced sucrose addition, likely due to its own high sugar content derived from barley malt. Snapping force determined with a texture analyzer decreased proportionally to sucrose reduction and MBF addition, indicating a softer texture of the cookies. MBF significantly increased the total phenolic content (TPC) and antioxidant activity (AOA) of the cookies. The results of the sensory analysis showed that cookies with *Pilsen* MBF and *Amber* MBF had a pleasantly sweet and rich flavor, while the addition of *Black* MBF produced an exaggerated bitter flavor and a nutty roasted aroma. The results suggest that different types of brewer’s MBF can be successfully used to produce functional cookies with reduced sucrose addition.

## 1. Introduction

The use of barley (*Hordeum vulgare* L.) as a raw material for food production is very low (2–3%) compared to all other uses of this widely cultivated crop (feed, brewing malt, seed) [1,2]. Historically, barley flour has long been used as a partial substitute for wheat flour (WF) in the production of various cereal-based products, but its value has been mostly neglected. Only recently, since the adoption of health claim legislation on the health benefits of β-glucan, has barley been recognized as a crop for the production of “healthy food”. Both the European Food Safety Authority (EFSA) and the U.S. Food and Drug Administration (FDA) have approved health claims for barley β-glucan and soluble fiber from barley in terms of lowering blood cholesterol and reducing the risk of coronary disease [3,4]. In addition, the nutritional value of barley is complemented by significant amounts of insoluble fiber, phenolic substances, minerals, and vitamins [2]. Although barley is known to be used in various products such as bread, cookies, breakfast cereals, etc., barley is mainly used by humans through the consumption of beer and other barley malt-based beverages. In addition, malt-based products in the form of malt flour, extract, or syrup are commonly used in the production of baked goods, cookies, and confectionery [5].

However, the malting process leads to numerous changes in the composition, as well as in the functional and nutritional properties of the barley grain. Malting barley grain involves four major steps: steeping (increasing water content), germination (sprouting), kilning and/or roasting (heat treatment), and cleaning (removing rootlets and impurities). The most noticeable change during germination is an intense synthesis of hydrolyzing enzymes (amylases, proteases, and β-glucanase). These enzymes modify the barley grain to the desired level. Normally, only a small amount of starch is saccharified at this stage, but proteins are degraded to a greater extent (even up to 70% of hordeins can be hydrolyzed to amino acids and smaller peptides during malting) [6]. In addition, according to some authors 50–60% of β-glucan is also degraded [7,8,9]. All these changes are aborted by applying increased temperature in kilning and/or roasting stages. Here, the malted grain is dried to the final moisture content (3–4%), and color and flavor are developed [10]. The more intense color and flavor develop when higher temperatures are applied. Moreover, depending on the temperature, the synthesized enzymes may or may not be destroyed, resulting in non-diastatic or diastatic malt. Diastatic malt flour is commonly used in small amounts as an additive to improve amylolytic activity in the dough of baked goods that require fermentation. Non-diastatic malt extract is typically used to improve color and flavor in cookies and may contribute to the sweetness of the product because it contains significant amounts of sugar [1,11].

There is very little research on the effects of adding malted barley flour (MBF) on cookie quality as either unmalted barley flour or malt extract is typically used in biscuit manufacture. As far as we know, there are very few studies that have investigated the influence of malted barley flour on the quality of cookies, and all of them have used base malt and not specialty brewer’s malt [12,13,14]. According to some previous studies, MBF can be used as a substitute for WF in amounts up to 50% without significantly deteriorating the sensory properties of the cookies [12]. In this way, products with higher nutritional value can be obtained, as the malting process improves protein digestibility, reduces phytic acid content, and dissolves bound phenolic compounds, resulting in increased bioavailability of some vitamins (B and C) and minerals (calcium, copper, manganese, and zinc) [15,16]. In addition, phenolic compounds and Maillard reaction products contribute to increased antioxidant activity [9,17]. In contrast, there is some evidence that malt may have pro-oxidant activity in addition to antioxidant activity, mainly due to increased lipolytic activity [17,18]. In addition, significant amounts of acrylamide may be produced during kilning and roasting, which must be considered when using MBF as a WF supplement in the composite flours for cookie production [19].

Normally, wheat short-dough cookies (also called “sweet” cookies) are considered to be foods with low nutritional value, as their recipes have high fat and sugar content [20]. The consumption of this type of cereal-based product is very high worldwide, so it must be taken into account that increased sucrose intake is associated with various health disorders and diseases such as obesity, diabetes, and tooth decay [21]. Therefore, any attempts to increase the nutritional value of cookie products are welcome. One way to achieve this goal is to use different composite flours [22,23]. In yeast-leavened bakery products, the use of composite flours is very limited because a relatively high wheat gluten content is required to obtain a quality product, and the replacement of WF also reduces the gluten content in the flour blend [24]. However, this is not the case for short-dough cookies. Unlike bakery products, cookies are made with low-protein flour and do not require extensive gluten development. This fact allows the use of flours from grains other than wheat [25]. On the other hand, problems may arise when sucrose is replaced or reduced in the recipe because sucrose is not only a sweetener, but an ingredient that significantly affects the technological quality of cookies [26]. The aim of this study was to investigate the effects of replacing part of the WF with three different types of brewer’s MBF with a simultaneous reduction of sucrose in the recipe on the physical and sensory properties of short-dough cookies. This study is a continuation of the previously conducted study in which only the influence of *Amber* MBF was investigated [27].

## 2. Results and Discussion

### 2.1. Physicochemical Analyzes of Malt and Composite Flour

Three different types of brewer’s MBF were used as partial substitutes for plain WF in a recipe for short-dough cookies: enzymatically active *Pilsen*, low-enzymatically active *Amber*, and non-enzymatically active *Black* malt (Slavonija slad d.o.o., Nova Gradiška, Croatia; Boortmalt, Antwerp, Belgium). The composite flours were prepared in MBF:WF ratios of 20:80, 40:60, and 60:40. The control sample was 100% WF.

#### 2.1.1. Reducing Sugar Content

During the malting process, some starch is broken down into various short-chain sugars and dextrins, many of which have reducing potential. Therefore, to accurately determine the reducing sugars in malt, one must know their exact molecular weight distribution. This is usually done using high-performance size-exclusion chromatography (HPSEC) [28,29]. Since in this study the Schoorl [30] method was used to determine the total reducing sugars and the exact molecular composition of the starch hydrolysis products is not known, all results are expressed on a maltose basis since the dominant reducing sugar in malt is maltose. Thus, the results obtained represent a reduction potential rather than the exact mass fraction of reducing sugars, which is sufficient for comparing different malts.

The content of reducing sugars in WF and MBF was determined as shown in Table 1. The results showed that the content of reducing sugars in MBF was significantly higher (*p* < 0.05) than in WF (0.43 g/100 g). The content of reducing sugars in *Pilsen* MBF was 7.75 g/100 g, which is in agreement with some previous studies, in which the content of reducing sugars in different base malts kilned under approximately similar conditions (maximum temperature about 80 °C) was 4.71–8.41% [31]. The reducing sugar content in *Amber* MBF was 17.05 g/100 g, and the highest content was found in *Black* MBF (61.02 g/100 g). According to the product specification of the malt producer, kilning of the base *Pilsen* malt takes place at temperatures ranging from 50 °C at the beginning to 80–87 °C at the end of the kilning process. The specialty malts *Amber* and *Black* are produced by roasting kilned base malt in a revolving roasting drum at 100–150 and 230 °C, respectively [32,33,34]. The high content of reducing sugars in MBF is the result of the enzymatic hydrolysis of starch and thermal dextrinization during the final step of malt production. The predominant sugar in the malt is maltose, followed by maltotriose and glucose, while the content of dextrins increases significantly with increasing temperature in the kilning/roasting stage [35,36]. Treatment of starch at high temperatures for a long period of time results in dark-brown dextrins. This is particularly evident in roasted *Black* malt, which is dominated by pyrodextrins [29,37].

#### 2.1.2. Solvent Retention Capacity (SRC) of Composite Flours

**Figure 1:**
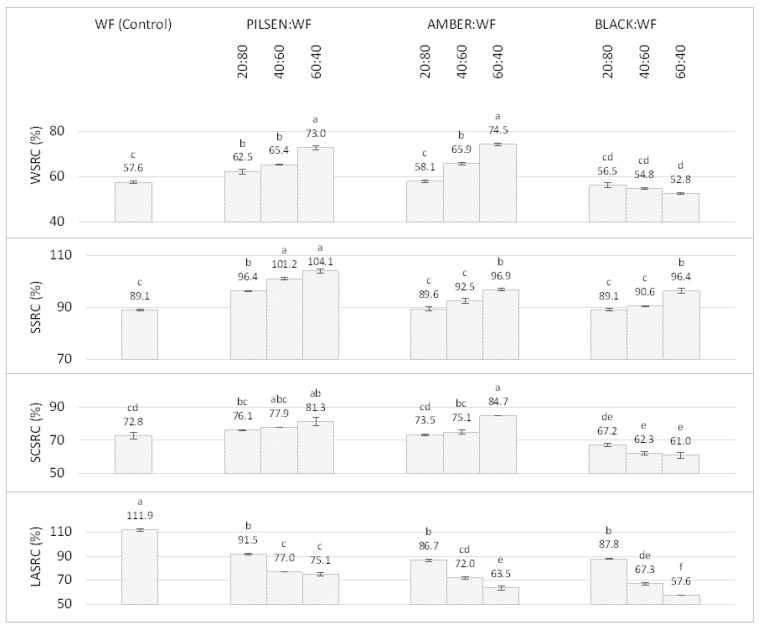
Solvent retention capacity (SRC) of malted barley and wheat composite flours. WSRC, SSRC, SCSRC, and LASRC denote water, sucrose, sodium carbonate, and lactic acid SRC, respectively. WF—Wheat flour. The values are Mean ± SD (*n* = 3). Different letters (a–f) indicate statistically significant differences (*p* < 0.05).

SRC is an assay based on monitoring the swelling potential of various flour-polymer networks (gluten, damaged starch, and pentosan) in selected individual diagnostic solvents. SRC with a lactic acid solution (LASRC) refers to swelling of glutenins, sodium carbonate to damaged starch (SCSRC), and concentrated sucrose solution (SSRC) to arabinoxylan. When only water is used as a solvent, the SRC results refer to all polymer components of the flour together (WSRC) [38]. The SRC values of all composite flours are shown in Figure 1.

As the proportion of *Pilsen* MBF and *Amber* MBF in the composite flour increased, the WSRC, SSRC, and SCSRC values increased significantly (*p* < 0.05). This may be attributed to the higher total absorption capacity of composite flours due to polymer modification during malting, increased solubility, and starch damage, as well as higher fiber content (arabinoxylan) and higher β-glucan content in MBF compared to WF [39,40]. The opposite effect was observed with LASRC. The addition of all three types of MBF caused a significant decrease (*p* < 0.05) in LASRC values. LASRC values decreased from 119.9% in the control WF to 75.1, 63.5, and 57.6% in the mixtures with 60% addition of *Pilsen*, *Amber*, and *Black* MBF, respectively. This was to be expected as the gluten content of composite flour decreased significantly with increasing substitution of MBF for WF [41]. The addition of *Black* MBF resulted in a significant decrease in WSRC, LASRC, and SCSRC due to the thermal decomposition of proteins and starch, which led to a decrease in their swelling capacity. SSRC increased from 89.1% in WF to 96.4% in composite flour with 60% *Black* MBF. This may be attributed to the resistance of arabinoxylans to roasting and the retention of their swelling potential. Similarly, when the effects of roasting on the chemical composition of the barley grain were studied, there was no change in the total dietary fiber content or their soluble and insoluble fractions [42].

#### 2.1.3. Pasting Properties of Composite Flours

Evaluation of the pasting properties is a widely used method for determining viscosity changes during the heating and cooling phases of flour (starch) water slurries.

A great deal of information about the gelatinization and liquefaction processes of starch, as well as amylolytic activity, can be obtained from the pasting curve (amylogram) [43].

**Figure 2:**
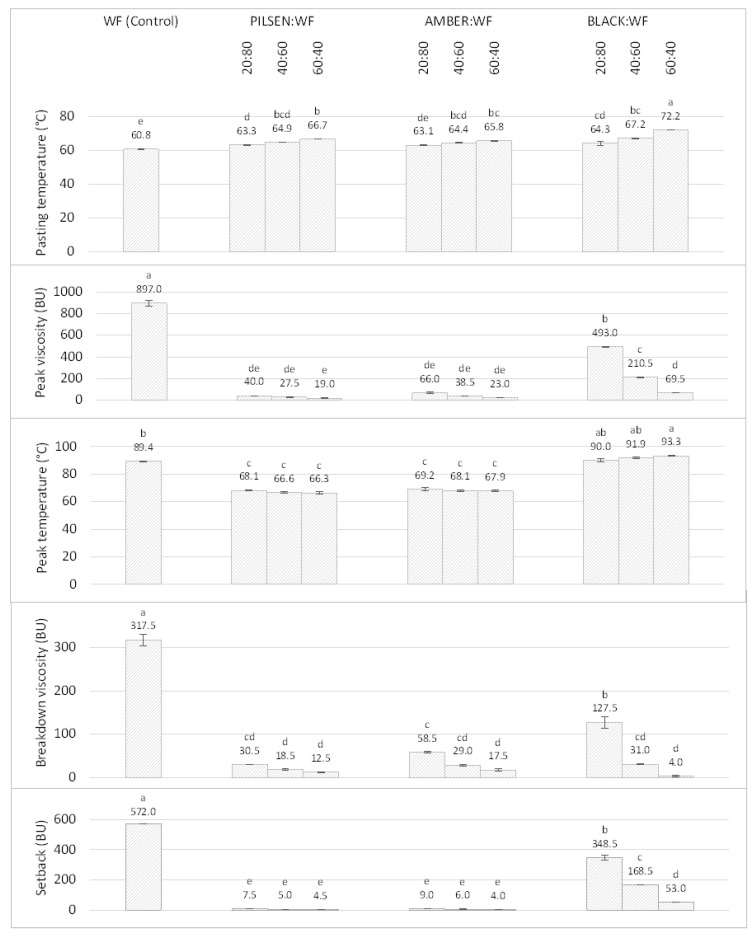
Pasting properties of malted barley and wheat composite flours. WF—Wheat flour. The values are Mean ± SD (*n* = 2). Different letters (a–e) indicate statistically significant differences (*p* < 0.05).

In this study, the Brabender Micro Visco-Amylo-Graph was used to investigate pasting properties of the MBF:WF slurries. As can be seen in Figure 2, the peak viscosity (897.0 BU), breakdown viscosity (317.5 BU), and setback (572.0 BU) were significantly higher for WF than for the MBF:WF composite flours (*p* < 0.05). The peak viscosity values of composite flours with *Pilsen* MBF were 40.0, 27.5, and 19.0 BU for MBF:WF ratios of 20:80, 40:60, and 60:40, respectively. The low viscosity values of the *Pilsen* MBF:WF composite flour slurries were primarily due to the increased amylolytic activity induced by malting. The effect of starch degradation of *Pilsen* MBF is probably less significant since the starch content of base malts is only about 5% lower than that of unmalted barley grain and α-amylase activity is significantly increased during germination [44]. Compared with *Pilsen* MBF, peak viscosity values increased slightly (66.0, 38.5, and 23.0 BU) when *Amber* MBF was used in the composite flour. It can be assumed that despite the elevated temperature during roasting of *Amber* malt, a small amount of enzymatic activity remains during the roasting process [45], resulting in a significant decrease in the peak viscosity of MBF:WF composite flour.

When *Black* MBF was used as a partial replacement for WF, the peak viscosity was lower than for 100% WF, but significantly higher than for the *Pilsen* and *Amber* MBF:WF composite flours. The peak viscosity values of composite flours with *Black* MBF were 493.0, 210.5, and 69.5 BU for MBF:WF ratios of 20:80, 40:60, and 60:40, respectively. In contrast to the *Pilsen* and *Amber* MBF:WF composite flours, the decrease in viscosity due to the addition of *Black* MBF was entirely due to changes in the chemical composition of the malt during the roasting process. Roasting inactivates the enzymes, but at the same time causes thermal degradation of the starch [46,47]. Therefore, the viscosity of slurries containing *Black* MBF depends on the amount of WF starch in the flour, and it was expected that the addition of *Black* MBF would decrease the viscosity proportionally to its addition.

Incorporation of all three types of MBF resulted in an increase in pasting temperature from 60.8 °C at 100% WF to 72.2 °C at MBF:WF ratio of 60:40, which is consistent with some previous studies in which pasting temperature was negatively correlated with peak viscosity [48]. Because diastatic *Pilsen* MBF and low-diastatic *Amber* MBF had extremely low peak viscosity, unlike WF, the peak temperature was also lower. The opposite effect was observed when *Black* MBF was used. The addition of *Black* MBF to composite flour resulted in an increase in peak temperature from 80.4 °C at 100% WF to 93.3 °C at MBF:WF ratio of 60:40. This can be explained by the absence of enzymatic activity and the high content of various sugars and maltodextrins in *Black* MBF, which cause a decrease in water activity and an increase in the stability of the amorphous regions of the starch granules in a starch suspension. Similar results were observed in other studies investigating the influence of sucrose and maltodextrin on the pasting properties of starch suspensions [49,50,51].

### 2.2. Physical Properties of Cookies

#### 2.2.1. Dimensional and Textural Properties of MBF:WF Cookies

The cookies were prepared according to AACC International Method 10-50.05 [30]. Control cookies were made from 100% WF and composite cookies were made from flours containing MBF and WF in the ratio of 20:80, 40:60, and 60:40. The addition of sucrose was reduced to 66.6% of the original amount of the cookie recipe with MBF and WF in a 20:80 ratio, 33.3% for the 40:60 ratio, and 0% for the 60:40 ratio. To study only the effects of reduced sucrose addition, WF cookies were also made with 66.6, 33.3, and 0% sucrose of the original amount in the recipe. Reducing the sucrose content also reduced the total solids content in the dough recipe. In addition, composite flours with the addition of MBF showed a significantly increased water absorption capacity. To avoid problems with mixing and dough development, the amount of water in the recipe was adjusted proportionally to the WSRC results and the amount of sucrose reduction. In this way, all samples had about the same water content (3–4%). The exact recipe for making the cookies can be found in Section 3.

**Figure 3:**
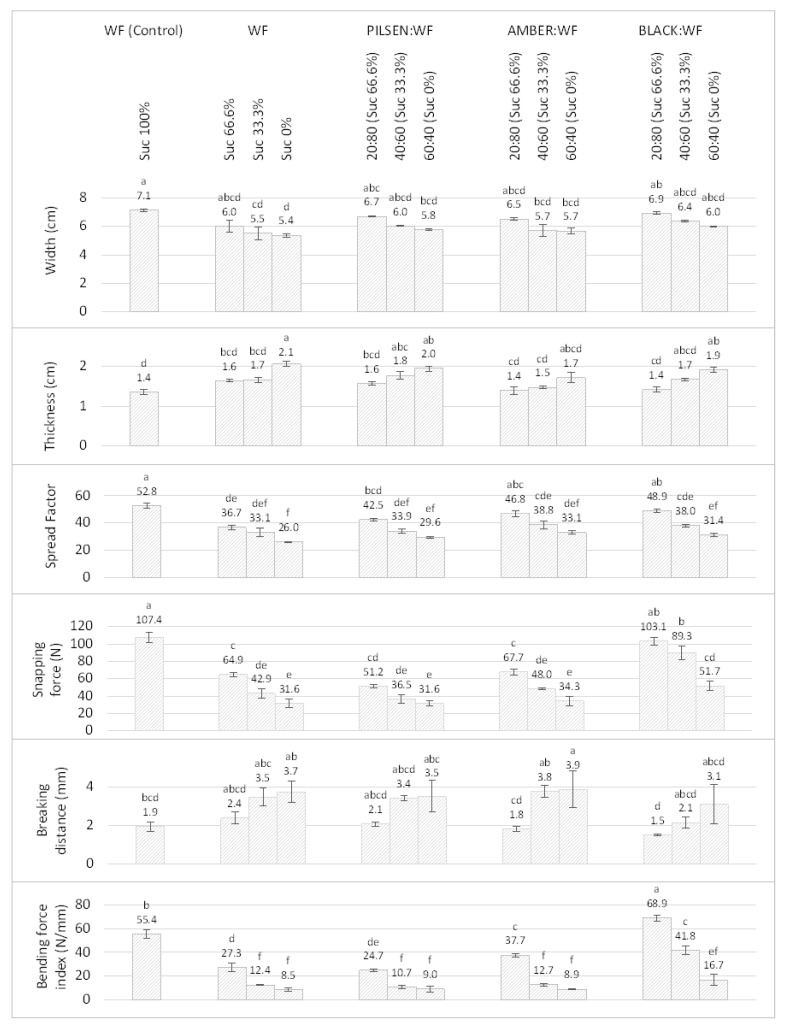
Dimensional and textural properties of cookies made from malted barley and wheat composite flours with reduced sucrose addition. WF—Wheat flour. Suc%: Percentage of added sucrose compared to standard recipe. The values are Mean ± SD (*n* = 6). Different letters (a–f) indicate statistically significant differences (*p* < 0.05).

Sucrose not only provides a sweet taste, but also affects the technological properties of cookies. In high concentrations, as in the short-dough cookie recipe, sucrose acts as an anti-plasticizer, preventing or reducing the development of gluten [51]. The development of gluten during the mixing of cookie dough should be minimal, just enough to allow the dough to form. Excessive development of gluten is undesirable and causes difficulties in sheeting and molding cookie dough. In addition, high concentrations of sugar, especially sucrose, prevent starch gelatinization during baking, leaving starch granules in baked cookies more or less in their native form [52]. Sugar makes cookies firm and brittle by controlling hydration and attempting to disperse protein and starch molecules, preventing the formation of a continuous gluten matrix and facilitating the formation of a continuous phase of glassy sugar syrup with embedded starch particles [53]. The sugar in the cookies reduces the viscosity of the dough, and during baking, the undissolved sugar gradually dissolves, contributing to the spread of the cookies. When the cookies cool, the sugar crystallizes and acts as a hardener [54]. For this reason, the sugar significantly affects the dimensions and texture of the cookies. Therefore, in order to control the change in the dimensions of the cookie, i.e., its spreading, it is necessary to regulate the sugar concentration in the dough and the development of gluten during mixing. The results of the dimensional changes and textural properties of the cookies are shown in Figure 3.

In this study, with the reduction of sucrose content in the recipe, the thickness of WF cookies increased and the width and spread factor decreased. The thickness of WF cookies was 1.4, 1.6, 1.7, and 2.1 cm, while the width was 7.1, 6.0, 5.5, and 5.4 cm with 100, 66.6, 33.3, and 0% added sucrose, respectively. Although variations in cookie dimensions were not statistically significant at 66% sucrose content (*p* < 0.05), increased height and decreased width resulted in a significant reduction in cookie spread (from 52.8 for standard WF cookies to 26.0 for cookies without added sucrose). The results obtained confirmed that when the sucrose concentration was reduced, some degree of gluten development occurred, reducing the spread of the cookies. These results are in agreement with previous results obtained by Pareyt et al. when the influence of sucrose content on the structural and textural properties of sugar-snap cookies was studied [21]. In addition, the cookies with reduced sucrose addition were irregularly shaped, as indicated by the increased standard deviations of the measured dimensions compared to the standard WF cookie.

When MBF was used in the cookie formulation with a simultaneous reduction in sucrose content, a similar trend in dimensional changes was observed. However, the intensity of these changes was lower compared to WF cookies with reduced sucrose content. The width of the composite cookies with an MBF:WF ratio of 20:80 and a sucrose content of 66.6% of the original formulation decreased slightly, but this decrease was not statistically significant compared to the control WF cookie (*p* < 0.05). The width of the cookies was 6.7, 6.5, and 6.9 cm for *Pilsen*, *Amber*, and *Black* MBF:WF (20:80), respectively. It can be concluded that the sugars from MBF compensated for the lack of sucrose to some extent, so the reduction in sucrose did not significantly affect the width of the cookies. A further increase in MBF content significantly reduced the width of the cookies. Using a 20:80 ratio of all MBF:WF, there were no significant differences in the height of the composite and control WF cookies, although the addition of sucrose was reduced to 66.6% of the original formulation. The height of the *Amber* and *Black* MBF:WF composite cookies did not increase significantly even at a ratio of 40:60. The spread factor decreased significantly for 20:80 *Pilsen* MBF:WF cookies and 40:60 *Amber* and *Black* MBF:WF, compared with WF cookies. Agrahar-Murugkar et al. also reported that the thickness of the cookies increased and the spread of the cookies decreased when malted finger millet flour was incorporated into the composite flour [55]. Since sucrose is the most effective sugar in regulating the mechanisms involved in the production of cookies, and only a small portion of malt sugar is sucrose, it can be assumed that the same amount of malt sugar does not have the same effect as the same amount of sucrose. A similar effect on cookie dimensions was found by Kweon et al. in their study on the effect of replacing sucrose with other types of sugar (glucose, fructose, xylose) on cookie quality [26].

The evaluation of the textural properties of the cookies was performed using a texture analyzer, and the three-point bend test was used. Considering only the snapping force and the breaking distance, it is not possible to accurately estimate the hardness of the cookies, since they do not all have the same thickness and their elasticity must be taken into account. In fact, some cookies break almost immediately, while others take a certain amount of bending time to break. Therefore, the maximum snapping force is always lower for the cookies that break immediately. Therefore, in this study, the *bending force index* parameter was used as the main indicator of hardness, since it more accurately describes the overall texture (hardness) of the cookies [25,52]. As can be seen in Figure 3, the textural properties of the cookies were significantly affected by the addition of sucrose and/or MBF. The reduced sucrose addition increased the breaking distance, decreased the snapping force, and consequently reduced the bending force index (*p* < 0.05), which was consistent with the study of Maache-Rezzoug et al. [54]. These results confirmed that a continuous glassy sucrose–water matrix significantly contributed to the texture of the cookies [56]. The snapping force, breaking distance, and bending force index of the *Pilsen* MBF:WF cookies indicated a softer cookie texture, suggesting that the addition of this type of MBF may not compensate for the changes caused by the reduced sucrose addition. This was to be expected since *Pilsen* malt is a base malt that is kilned at relatively low temperatures and produces little simple sugar during malting. To our knowledge, there are few studies that have examined the impact of malted barley flour on cookie quality. These studies reached similar conclusions. Sharma et al. reported that MBF:WF composite cookies became softer and more crumbly and explained this phenomenon by the increased fiber and decreased gluten content in the composite flour [12]. In the study by Agrahar-Murugkar et al., they reduced cutting strength and cohesion of the cookies was also attributed to the lower gluten content in the composite flour with malted finger millet flour [55].

However, when specialty malts with higher sugar content were used, this softening effect was reduced. The breaking force index for *Amber* MBF:WF (20:80) composite cookies with 66% added sucrose (32.7 N/mm) was lower than for the control WF cookie (55.4 N/mm), but significantly higher than for WF cookies with 66% added sucrose (27.3 N/mm). When *Black* MBF was used, the breaking force index for the 20:80 MBF:WF ratio was even higher (68.9 N/mm) than for the control WF cookie. This can also be explained by the high sugar content of *Amber* and *Black* MBF, which attenuates the effect of the reduced sucrose addition.

#### 2.2.2. Color of MBF:WF Cookies

In this study, color was measured using computer vision, an indirect technique that quantifies the amount of light reflected from the surface of the cookies. Samples were captured with a flatbed scanner and the digitized images were processed with *ImageJ* software using the Color Histogram plugin. The CIEL*a*b* color model was used to evaluate the cookie colors, as this model has the largest color range and mimics human vision. The parameter L* represents the luminance or lightness of the sample and ranges from 0 (black) to 100 (white). The chromatic components a* and b* can range from −128 to 127. The parameter a* stands for the green-red and b* for the blue–yellow axis of the color space.

**Figure 4:**
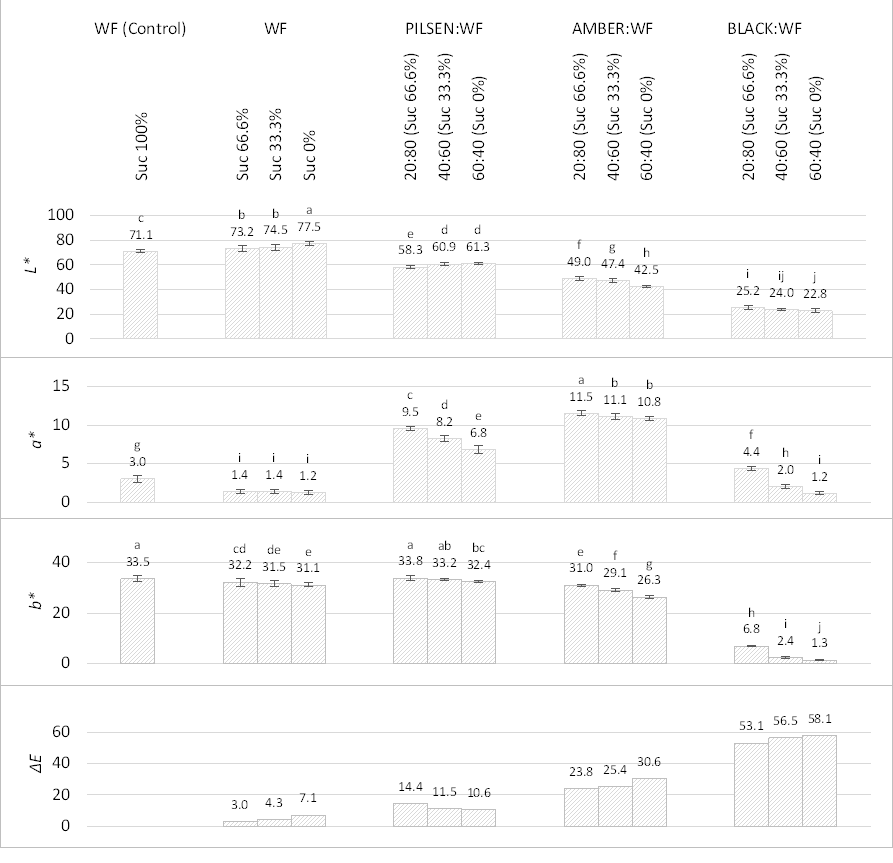
Color of cookies made from malted barley and wheat composite flours with reduced sucrose addition. WF—Wheat flour. Suc%: Percentage of added sucrose compared to standard recipe. Color difference (Δ*E*) refers to the difference between the sample and the control WF cookie. The values are Mean ± SD (*n* = 6). Different letters (a–j) indicate statistically significant differences (*p* < 0.05).

Besides the change in dimensions and texture, the change in color is one of the most pronounced changes that take place when cookies are baked. The color of the cookies is largely determined by the sugar content in the flour, the baking time and temperature, and the amount of colored ingredients in the cookie recipe. The results showed that the color of the cookies was significantly affected by the amount of sucrose added and the MBF content in the composite flour (Figure 4). A decrease in sucrose content caused the color of WF cookies to become paler. A significant increase in L* value (71.1 to 77.5) and decrease in b* value (33.5–31.1) were observed when sucrose was omitted from the recipe (*p* < 0.05). As expected, the control WF cookies were darker and had a more pronounced yellowish–golden color than the WF cookies with reduced added sucrose. It is well known that sucrose contributes to the color development of the cookies due to the chemical reactions induced by the heat during baking. Color development can be attributed to two processes involving sugar: Maillard reactions with amino acids and caramelization. Sucrose itself is not a reducing sugar, but during baking, some sucrose is hydrolyzed into glucose and fructose and thus can participate in Maillard reactions and browning of cookies [57]. The hydrolysis of sucrose is limited due to the low moisture content in cookies, but just sufficient to contribute to color development. It should be noted that thermal dextrinization of starch is another important reaction in color formation in cookies [52].

The color of the cookies depended on the type of MBF used and its initial color, affecting the color of the cookies proportionally to its addition. The color of the malt comes from the barley malting process and depends mainly on the temperature during kilning and/or roasting [10]. The *Pilsen* MBF was the palest of all three MBFs, the *Amber* MBF was darker with a more intense amber (reddish-gold) color, while the Black MBF was very dark, almost black. Accordingly, the lightness (L*) decreased with the addition of MBF in the following order: *Pilsen* > *Amber* > *Black* MBF because the specialty malts are rich in melanoidins. Interestingly, the L* value for *Pilsen* MBF:WF was higher in the 60:40 ratio than in the 20:80 ratio. This can be explained by the fact that *Pilsen* MBF is quite light in color and no sucrose was added in the 60:40 ratio, so *Pilsen* MBF’s own sugar could not fully compensate for the lack of sucrose in the formulation. The a* color component had the highest values when *Amber* MBF was used in the formulation, resulting in a reddish amber color of the cookies. b* values, indicating a yellow color component, were highest in *Pilsen* MBF:WF cookies. The very low L*a*b* values of the *Black* MBF:WF cookies were the result of a very dark, almost black cookie color. The overall color differences (Δ*E*) between the control WF and the composite cookies were well above five, indicating color differences that can be easily perceived by the average consumer [58].

### 2.3. Total Phenolic Content (TPC) and Antioxidant Activity (AOA) of MBF:WF Cookies

**Figure 5:**
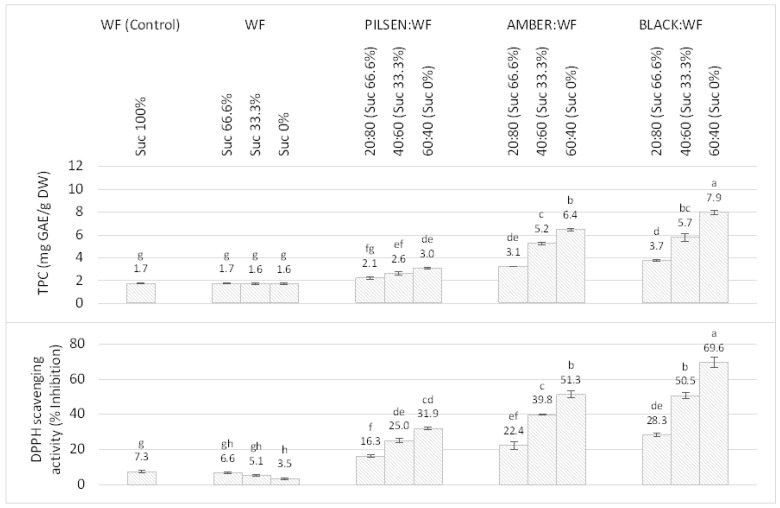
Total phenolic content (TPC) and antioxidant activity (AOA) of cookies made from malted barley and wheat composite flours with reduced sucrose addition. WF—Wheat flour. Suc%: Percentage of added sucrose compared to standard recipe. The values are Mean ± SD (*n* = 3). Different letters (a–h) indicate statistically significant differences (*p* < 0.05).

TPC and AOA of the composite cookies were significantly higher than those of the WF cookies (*p* < 0.05). As shown in Figure 5, the TPC was 2.1, 2.6, and 3.0 mg GAE/g in *Pilsen*, 3.1, 5.2, and 6.4 mg GAE/g in *Amber*, and 3.7, 5.7, and 7.9 mg GAE/g in *Black* MBF:WF cookies with MBF:WF ratios of 20:80, 40:60, and 60:40, respectively. Simultaneously with the increase in TPC, AOA also increased. *Black* MBF showed the highest DPPH scavenging potential with inhibition up to 69.6% at a 60:40 MBF:WF ratio. This is consistent with numerous previous studies suggesting that the malting process releases a certain amount of bound phenolic compounds and increases AOA [17].

TPC and AOA of barley malt have been reported to increase both during germination and kilning/roasting stage [59,60]. However, the total AOA of malt not only depends on the phenolic compounds released, but also increases due to the development of the Maillard reaction and caramelization products. The products of Maillard reactions exhibit radical scavenging, metal chelating, and reducing power properties, thus contributing to the overall antioxidant capacity [61]. This suggests that darker malts kilned or roasted at higher temperatures have a higher AOA regardless of TPC [62,63].

No significant change in TPC was observed in WF cookies with reduced sucrose addition compared to the control WF cookies. The AOA decreased with decreasing sucrose content from 7.3 to 3.5% in WF cookies without added sucrose, suggesting that the Maillard reaction products and the caramelization products formed from sucrose after thermal hydrolysis during baking have some antiradical properties and reducing potential, as reported by other authors [62,63,64,65]. Given the results obtained from the AOA of MBF:WF cookies, it can be concluded that the inclusion of MBF in the cookie recipe can improve the nutritional properties and health benefits of the product. However, there is some evidence that malt, especially the darker malts, may have a pro-oxidant effect in addition to the AOA, mainly due to increased lipolytic activity [17,18], so this should also be considered. In addition, some researchers suggest that the products of the Maillard reaction contribute to pro-oxidant activity through their ability to scavenge oxygen radicals and chelate metals [61].

### 2.4. Sensory Evaluation

Table 2 summarizes the results of the sensory evaluation (nine-point hedonic scale) of the composite cookies and includes scores for color, shape, texture, odor, taste, and overall acceptability. *Black* MBF:WF composite cookies had the lowest sensory scores, followed by WF cookies with reduced sucrose addition.

The control WF cookies had the highest sensory ratings with an overall acceptance score of 7.6, placing this sample between “like moderately” and “like very much” on the nine-point hedonic scale. The scores for all sensory attributes decreased with the reduction of sucrose content in the recipe of WF cookies. The discoloration and irregular shape of the cookies were easily noticed by the sensory evaluators. The texture of the cookies with reduced sucrose content was described as too chewy and the pleasant aroma was reduced compared to the control WF cookies. The panelists were instructed to pay particular attention to the sweetness of the cookies when evaluating taste. The lack of sweetness was readily apparent even in cookies with 66.6% added sucrose from the original recipe. This observation is consistent with the results of Biguzzi et al. in which a 16% reduction in sugar content affected the likability score of the cookies [66]. It should also be mentioned that some panelists found the control WF cookies too sweet.

Overall acceptability scores for *Pilsen* and *Amber* MBF:WF composite cookies with a 20:80 MBF:WF ratio were 6.6 and 7.1, respectively, which was lower than WF cookies but not statistically significant (*p* < 0.05). Further increasing the addition of the *Pilsen* and *Amber* MBF addition significantly deteriorated liking scores of the composite cookies. The texture of the *Pilsen* MBF:WF cookies was judged to be quite soft, and several assessors emphasized the lack of sweetness of these cookies, even at a 20:80 MBF:WF ratio. In contrast to *Pilsen* MBF:WF, the lower sweetness of *Amber* MBF:WF cookies was not readily apparent at a 20:80 MBF:WF ratio and 66.6% sucrose content. The panelists noted a rich flavor and a pleasantly sweet caramel aroma. Therefore, the rating for odor and taste was quite high, 7.3 and 7.4, respectively. The color of these cookies was described as attractive with an interesting dark amber hue. The texture was rated as slightly crunchy. *Black* MBF:WF cookies received low scores even at a 20:80 MBF:WF ratio. Overall acceptability scores ranged from 4.7 for composite cookies with a 20:80 MBF:WF ratio to 1.5 at a 60:40 MBF:WF ratio. The only acceptable score was for cookie shape appearance, which was relatively regular at this ratio and not significantly different from the control WF cookies. Panelists highlighted the unpleasantly bitter, “overly toasted” aroma and taste, as well as the unpleasant odor that came from the pyrolysis compounds. The texture was described as hard and crumbly. This was to be expected since the *Black* MBF had a fairly pronounced “toasted” aroma, but it was used in the experiment in the same amounts as other MBFs to compare its influence on the physicochemical properties of the cookies. In future studies, the use of *Black* MBF in very small amounts (e.g., <5%) should be considered, especially to obtain a darker cookie color.

Alka et al. reported that even with the addition of 60% MBF, relatively acceptable cookies and high sensory scores could be obtained [14]. In the study of El-Hadary et al. it was shown that cookies with 30% MBF had good organoleptic properties close to those of 100% WF cookies [13], and in the work of Sharma and Chopra a 50% replacement of WF by MBF had no deteriorating effect on the sensory quality of the cookies [12]. However, in contrast to our study, sucrose addition was not reduced in all these studies. In our study, we concluded that acceptable cookies could be made by adding 20% *Pilsen* and *Amber* MBF and simultaneously reducing sucrose to 66.6% of the original standard recipe.

### 2.5. Limitations to the Study

As mentioned above, the use of *Black* MBF in large quantities has a negative effect on the quality of the cookies. Although we knew that *Black* MBF in such amounts would have a negative effect on sensory properties, we wanted to compare its effects on the technological properties of cookies with *Pilsen* and *Amber* MBF. Future research should not completely exclude the use of this malt, but should investigate its effects with a significantly lower addition. In addition, intensive roasting of malt can produce significant amounts of acrylamide. This is particularly important in the context of consumer health and legislation that has recently recognized this problem and mandated maximum levels of acrylamide in various foods and thus in various types of baked goods and snacks, including cookies. Therefore, when investigating options for the inclusion of dark types of MBF in product formulation, a determination of acrylamide should also be made. Another limitation of this study is that no qualitative analyzes of sugars were performed. In the context of this study, this would be very useful information to better clarify the effects of different malt sugars on cookie quality. Since the results of this study did not provide information on the individual effects of the variables (MBF substitution level and reduced sucrose content) on the quality of the cookies, our future research will focus on optimizing the MBF:WF cookie formulation by conducting a Mixture Design of Experiments (DOE).

## 3. Materials and Methods

### 3.1. Materials

Commercial cookie plain WF (Tena-Žito Ltd., Đakovo, Croatia) and three different types of brewer’s MBF were used for this study: enzymatically active *Pilsen*, low-enzymatically active *Amber,* and non-enzymatically active *Black* malt (Slavonija slad d.o.o., Nova Gradiška, Croatia; Boortmalt, Antwerp, Belgium). The proximate composition of WF and MBF was provided by the manufacturers and showed in Table 3. The other ingredients were sucrose, shortening (Zvijezda d.d., Zagreb, Croatia), sodium chloride (NaCl), and sodium bicarbonate (NaHCO_3_) from a local market.

### 3.2. Methods

#### 3.2.1. Reducing Sugar Content in Flour

The Schoorl method was used to determine the reducing sugar content according to the AACC International Method 80-68.01 [30]. In this method, the amount of reduced copper from Fehling’s solution determined after iodometric titration with sodium thiosulfate solution is proportional to the content of reducing sugars in the flour sample. Since the exact molecular composition of the starch hydrolysis products was not known, all results were expressed on a maltose basis, since the dominant reducing sugar in malt is maltose. Three replicate measurements were made for each sample.

#### 3.2.2. Solvent Retention Capacity (SRC) of Composite Flours

The SRC values of all composite flours were obtained using the AACC International Method 56-11.02 [30]. Four different SRC solvents were used: water, 50% sucrose, 5% sodium carbonate, and 5% lactic acid solution. The sample (5 g) was dissolved with each solvent (50 mL) for 20 min and the retention capacity was calculated from the weight of the pellet remaining after centrifugation, decantation, and draining. Three replicate measurements were made for each sample.

#### 3.2.3. Pasting Properties of Composite Flours

The pasting properties of MBF:WF composite flours were evaluated using the Micro Visco-Amylo-Graph (Brabender OGH, Duisburg, Germany). A suspension of 15 g (14% w. b.) and 100 mL distilled water was heated from 30 to 92 °C at 5 °C/min rate, then held constant at 92 °C for 5 min, cooled to 50 °C at 5 °C/min and finally held at 50 °C for 1 min. The share of 250 min^−1^ was held constant throughout the analysis. The pasting temperature, peak temperature, peak viscosity, breakdown, and setback viscosity were recorded. Viscosity parameters were expressed in Brabender units (BU). Two replicate measurements were made for each sample.

#### 3.2.4. Production of the Composite Cookies

The cookies were prepared according to the AACC International Method 10-50.05 [30] with some modifications. The exact recipe can be found in Table 4. Shortening, sucrose, salt, and sodium bicarbonate were placed in a mixing bowl of an electronic mixer (Gorenje MMC800W, Velenje, Slovenia) and mixed at slow speed for 3 min. Water was then added and mixed for another minute at low speed and another minute at medium speed. Finally, the composite flour was added and mixed for 2 min at the lowest speed. After mixing, the dough was rounded, placed in a plastic bag, and allowed to rest in the refrigerator at 8 °C for 30 min. After cooling, the dough was flattened to a thickness of 7 mm using a dough roller. The dough pieces were cut out with the cylindrical mold into a round shape of 60 mm diameter and placed in a baking tray lined with baking paper. Baking was performed in a convection oven (Wiesheu Minimat Zibo, Wiesheu GmbH, Grossbottwar, Germany) for 12 min at 205 °C. Dextrose, which is usually added in this method to help develop the brown color, was not used to better assess the effects of sucrose and malt sugars. To avoid mixing and dough development problems, preliminary baking tests were conducted to adjust the amount of water in the recipe proportionally to the WSRC results and the extent of sucrose reduction. The cookies were made in triplicate batches.

#### 3.2.5. Physical Analysis

The dimensions of the cookies were measured according to the AACC International Method 10-50.05 [30]. The total width of six cookies stacked next to each other was measured. Then, each cookie was rotated 90° and measured again. The average width was calculated and divided by six to obtain W (cm). Six cookies were placed on top of each other and their total height was measured. Then, the cookies were randomly rearranged and the height was measured again. The average height of six cookies was divided by six to obtain T (cm). The spread factor was calculated as W/T multiplied by ten. Six sample cookies from every batch were measured.

After cooling at room temperature for three hours, the texture of the cookies was evaluated using the TA.XT2i Texture Analyzer (Stable Microsystems Ltd., Surrey, UK). The three-point bend-break test was used. The knife blade moved toward the cookie located between the two lower supports with a distance of 40 mm between them. The test was performed at a knife speed of 1 mm/s until the breaking point was reached. The snapping force (N) and the breaking distance (mm) were recorded and the ratio between them was calculated to obtain the bending force index (N/mm), which is a measure of the softness/hardness of the cookie. Two sample cookies from every batch were evaluated.

#### 3.2.6. Color Evaluation

The color of the cookies was measured using computer vision as previously described in detail [25]. Samples were captured with a flatbed scanner EPSON Perfection^®^ V500 Photo (Epson America Inc., Los Alamitos, CA, USA) and the digitized images were processed with *ImageJ* software using the Color Histogram plugin [67]. The obtained results were converted from RGB to CIEL*a*b* values and then the CIEL*a*b* color model was used to evaluate the color of the cookie, since this model has the largest color range and mimics human vision [68]. The parameter L* represents the luminance or lightness of the sample and ranges from 0 (black) to 100 (white). The chromatic components a* and b* can range from −128 to 127. The parameter a* stands for the green–red and b* for the blue–yellow axis of the color space. The total color difference (∆*E*) between the control and sample cookies was calculated using the CIE76 color difference equation, which represents the Euclidean distance between two points in CIEL*a*b* space [58]. Two sample cookies from every batch were evaluated.

#### 3.2.7. Total Phenolic Content (TPC) and Antioxidant Activity (AOA)

One gram of the ground cookie sample was extracted with 10 mL of 80% methanol. The mixture was vortexed for 1 min, sonicated in an ultrasonic bath for 10 min, and shaken on an electronic shaker at 150 rpm for 2 h. After centrifugation at 3000× *g* for 10 min, the supernatant was collected and used for the determination of TPC and AOA.

For TPC determination, the extract (0.3 mL) was mixed with diluted (1:10) Folin-Ciocalteu reagent (1.5 mL) and mixed vigorously for 3 min. Then, a 6.0% Na_2_CO_3_ solution (1.5 mL) was added, the mixture was shaken, and left in a dark place for 90 min. The absorbance was measured spectroscopically at 760 nm. The TPC was calculated from the calibration curve of gallic acid as mg GAE/100 g dry weight [69].

AOA was evaluated using 2,2-diphenyl-1-picrylhydrazyl (DPPH). The reaction mixture was prepared using 0.1 mL of extract and 3.9 mL of DPPH methanol solution (0.1 mM). The mixture was shaken, left in the dark for 30 min, and absorbance was measured at 517 nm. The AOA was expressed as the percentage inhibition of the DPPH radical [70]. Three replicate measurements were made for each sample.

#### 3.2.8. Sensory Analysis

The sensory evaluation of the cookies was carried out by a panel of ten semi-trained evaluators with previous experience in sensory analysis, all of them employees and students of the Department of Cereal Technology of the Faculty of Food Technology Osijek. There were three men and seven women in the panel (median age = 24 years). The inclusion criteria for assessors were: normally consume this type of product, absence of any health problems that could affect the sensory evaluation, such as color vision disorders, anosmia and the like. The nine-point hedonic scale was used to evaluate individual sensory characteristics: color, shape, texture, odor, taste, and overall accessibility. The ratings were: dislike extremely (1), dislike very much (2), dislike moderately (3), dislike slightly (4), neither like nor dislike (5), like slightly (6), like moderately (7), like very much (8), and like extremely (9).

#### 3.2.9. Statistical Analysis

Experimental data were analyzed with analysis of variance (ANOVA) and differences between samples were tested with Tukey’s Honestly Significant Difference (HSD) test (*p* < 0.05). Statistical analysis was performed using XLSTAT software (Addinsoft, New York, NY, USA).

## 4. Conclusions

With the increasing production and rapid development of the brewing industry, there are many types of brewing malts on the market, which can also be used for making cookies. Brewing malts have a very wide range of different properties and can affect the development of cookies with a wide range of properties. Therefore, due to their different characteristics, the different types of malt require a different approach when developing a recipe for cookie production. The results of this study showed that by replacing WF with 20% *Amber* or *Pilsner* malt while reducing the addition of sucrose to 66.6% of the original standard recipe, the cookies retained similar qualitative characteristics to WF cookies made from a standard recipe. The MBF mitigated the effects of the reduced sucrose addition, likely due to its own high sugar content derived from barley malt. The addition of *Black* MBF in large quantities resulted in an unpleasant bitter aroma and taste, as well as poor technological properties of the cookies. Therefore, its use in much smaller quantities should be considered only if a dark color of the cookies is to be achieved.

Since barley malt has numerous nutritional and health-promoting properties (high TPC and AOA, high β-glucan content, improved protein digestibility), it can be concluded that the use of MBF in cookie production can reduce the use of sucrose and produce cookies with improved functional properties and increased nutritional value.

## Figures and Tables

**Table 1:** Content of reducing sugars in wheat flour (WF) and malted barley flours (MBF).

Flour	Reducing Sugar Content (g/100 g) ^1^
WF	0.43 ± 0.09 ^d^
Pilsen MBF	7.75 ± 0.21 ^c^
Amber MBF	17.05 ± 0.19 ^b^
Black MBF	61.02 ± 0.32 ^a^

^1^ The values are Mean ± SD (*n* = 3). Different letters (a–d) indicate statistically significant differences (*p* < 0.05).

**Table 2:** Sensory scores of cookies made from malted barley and wheat composite flours with reduced sucrose addition.

MBF:WF ^1^	Sucrose (%) ^2^	Color	Shape	Texture	Odour	Taste	Overall
WF (Control)							
0:100	100	7.9 ± 1.0 ^a 3^	7.9 ± 0.6 ^a^	7.2 ± 1.2 ^a^	7.3 ± 1.3 ^a^	7.6 ± 0.5 ^a^	7.6 ± 0.7 ^a^
WF							
0:100	66.6	6.7 ± 1.2 ^abc^	5.7 ± 1.7 ^bcd^	5.9 ± 0.8 ^abc^	6.0 ± 1.2 ^ab^	6.1 ± 0.6 ^abc^	6.1 ± 1.0 ^abc^
0:100	33.3	3.6 ± 0.9 ^def^	2.4 ± 1.3 ^ef^	3.1 ± 1.0 ^cde^	3.3 ± 1.3 ^cd^	3.0 ± 0.8 ^def^	3.0 ± 1.1 ^def^
0:100	0	2.1 ± 1.1 ^f^	1.6 ± 1.0 ^f^	1.6 ± 0.9 ^e^	2.1 ± 1.2 ^d^	1.7 ± 0.7 ^g^	1.5 ± 0.6 ^f^
PILSEN:WF							
20:80	66.6	6.6 ± 1.0 ^abc^	7.2 ± 1.1 ^ab^	6.5 ± 1.0 ^ab^	6.6 ± 1.0 ^a^	6.5 ± 0.8 ^ab^	6.6 ± 1.0 ^abc^
40:60	33.3	4.9 ± 1.0 ^bcde^	4.3 ± 0.7 ^cde^	5.1 ± 1.6 ^abcd^	5.1 ± 1.2 ^abc^	4.1 ± 0.6 ^cdef^	4.7 ± 0.9 ^cde^
60:40	0	3.0 ± 1.2 ^ef^	2.9 ± 1 ^ef^	3.1 ± 1.2 ^cde^	3.4 ± 1.5 ^bcd^	2.2 ± 1.1 ^fg^	2.6 ± 0.8 ^ef^
AMBER:WF							
20:80	66.6	7.3 ± 1.0 ^ab^	7.3 ± 0.7 ^ab^	7.0 ± 1.6 ^a^	7.3 ± 1.3 ^a^	7.4 ± 1.9 ^a^	7.1 ± 1.7 ^ab^
40:60	33.3	6.1 ± 1.9 ^abcd^	5.3 ± 1.3 ^bcd^	4.7 ± 1.3 ^abcd^	5.3 ± 1.3 ^abc^	5.1 ± 1.2 ^bcd^	5.1 ± 1.1 ^bcd^
60:40	0	4.4 ± 2.1 ^cdef^	3.6 ± 1.7 ^def^	3.3 ± 1.3 ^cde^	3.7 ± 1.3 ^bcd^	2.4 ± 1.2 ^efg^	3.3 ± 0.9 ^de^
BLACK:WF							
20:80	66.6	4.8 ± 1.5 ^bcde^	6.3 ± 1.4 ^abc^	4.4 ± 1.8 ^bcd^	3.4 ± 1.4 ^bcd^	4.3 ± 1.5 ^cde^	4.7 ± 1.3 ^cde^
40:60	33.3	2.7 ± 1.3 ^ef^	3.6 ± 1.0 ^def^	2.7 ± 1.4 ^de^	2.3 ± 1.2 ^d^	2.0 ± 0.8 ^fg^	2.7 ± 0.9 ^ef^
60:40	0	2.1 ± 1.6 ^f^	2.0 ± 0.9 ^ef^	1.6 ± 0.8 ^e^	1.5 ± 0.7 ^d^	1.4 ± 0.9 ^g^	1.5 ± 0.8 ^f^

^1^ MBF—Malted barley flour; WF—Wheat flour. ^2^ Percentage of added sucrose compared to standard recipe. ^3^ The values are Mean ± SD (*n* = 10). Mean values in the same column with different superscript letters (a–g) are significantly different (*p* < 0.05).

**Table 3:** Proximate composition of wheat flour (WF) and malted barley flour (MBF) (g/100 g d.m.).

Flour	Protein	Fat	Fiber	Carbohydrate	Ash
WF	10.6	1.6	0.71	86.5	0.57
Pilsen MBF	11.2	1.5	4.8	80.3	2.21
Amber MBF	11.1	1.4	4.9	80.5	2.09
Black MBF	10.8	1.4	5.1	80.6	2.15

**Table 4:** Formulation of MBF:WF cookies.

	WF (g) ^1^	MBF (g)	Shortening (g)	Sucrose (g)	NaCl (g)	NaHCO_3_ (g)	Water (mL)
WF (Control)	100	-	28.4	57.8 (100%) ^2^	0.9	1.1	21.0
WF	100	-	28.4	38.1 (66.6%)	0.9	1.1	20.4
	100	-	28.4	19.1 (33.3%)	0.9	1.1	19.2
	100	-	28.4	- (0%)	0.9	1.1	18.0
PILSEN:WF	80	20	28.4	38.1 (66.6%)	0.9	1.1	22.8
	60	40	28.4	19.1 (33.3%)	0.9	1.1	23.8
	40	60	28.4	- (0%)	0.9	1.1	26.6
AMBER:WF	80	20	28.4	38.1 (66.6%)	0.9	1.1	21.2
	60	40	28.4	19.1 (33.3%)	0.9	1.1	24.0
	40	60	28.4	- (0%)	0.9	1.1	27.1
BLACK:WF	80	20	28.4	38.1 (66.6%)	0.9	1.1	20.6
	60	40	28.4	19.1 (33.3%)	0.9	1.1	20.0
	40	60	28.4	- (0%)	0.9	1.1	19.2

^1^ MBF—Malted barley flour; WF—Wheat flour. ^2^ Percentage of added sucrose compared to standard recipe (100% sucrose = 57.8 g/100 g flour basis according to AACC Method).
